# Cobalt-Induced Magnéli Phase Transformation Towards Oxygen Evolution and Oxygen Reduction Reactions

**DOI:** 10.3390/ma19194099

**Published:** 2026-09-25

**Authors:** Tijana Milivojević, Maja Ranković, Jadranka Milikić, Stevan Stojadinović, Bojana Nedić Vasiljević, Danica Bajuk-Bogdanović, Maja Milojević-Rakić, Nemanja Gavrilov

**Affiliations:** 1 Centre for Ecotoxicological Research, Bulevar Šarla de Gola 2, 81000 Podgorica, Montenegro; tijana.milivojevic@ceti.me; 2 Faculty of Physical Chemistry, University of Belgrade, Studentski Trg 12–16, 11158 Belgrade, Serbia; maja.rankovic@ffh.bg.ac.rs (M.R.); jadranka@ffh.bg.ac.rs (J.M.); bojana@ffh.bg.ac.rs (B.N.V.); danabb@ffh.bg.ac.rs (D.B.-B.); gavrilov@ffh.bg.ac.rs (N.G.); 3 Faculty of Physics, University of Belgrade, Studentski trg 12–16, 11158 Belgrade, Serbia; sstevan@ff.bg.ac.rs

**Keywords:** Magnéli phase; cobalt; oxygen reduction, oxygen evolution, titanium suboxides

## Abstract

Hydrothermal deposition of cobalt on mixed-phase titanium suboxides, Ti_5_O_9_ and Ti_8_O_15_ (TSO), was performed under mild conditions. High-temperature treatment under a reducing atmosphere led to a phase reversal, contrary to the expected Co-doping into the TSO. Surface cobalt species led to “unshearing” of crystallite planes, reverting to Co-decorated rutile. SEM-EDX measurements confirmed homogeneous dispersion of cobalt on the surface amounting to 4.15 wt.%. Raman and FTIR measurements confirmed the presence of Co at the surface, albeit in oxidized form, converting the Co to Co_3_O_4_ under atmospheric conditions. Modification of TSO by Co significantly improved OER and, to a certain degree, ORR. The charge transfer resistance for OER diminished from 17.5 kΩ to 47.4 Ω, indicating excellent activity towards oxygen evolution, with the onset potential for OER shifting to 1.68 V vs. RHE. RRDE measurement revealed that upon Co-surface deposition, the mechanism of ORR changes only slightly towards the 4e^−^ branch and is still dominated by the 2e^−^ pathway, characteristic of TSO, due to low cobalt content, with an onset potential for ORR of 0.72 V vs. RHE.

## 1. Introduction

The design of efficient and durable electrocatalysts for oxygen-involving reactions is essential for the advancement of water electrolyzers, fuel cells, regenerative fuel cells, and rechargeable metal–air batteries [1,2,3]. In these technologies, the oxygen evolution reaction (OER) and oxygen reduction reaction (ORR) are among the most kinetically demanding electrode processes due to multistep proton-coupled electron transfer and the formation/cleavage of O–O bonds [1,2]. Their sluggish kinetics result in high overpotentials and substantial energy losses. Although IrO_2_ and RuO_2_ are considered benchmark OER catalysts, and Pt-based materials are the most active conventional ORR catalysts, their scarcity, high cost, and insufficient long-term stability restrict their large-scale application [1,2,3]. Consequently, considerable research has focused on earth-abundant transition-metal oxides that can catalyze one or both oxygen reactions.

Cobalt-based oxides and oxyhydroxides are promising non-precious electrocatalysts for oxygen reactions, particularly in alkaline media [4,5,6]. The accessible Co^2+^/Co^3+^/Co^4+^ redox transitions and the ability of cobalt centers to interact with oxygenated intermediates (OH*, O*, OOH*, and O_2_*) guide their activity [7,8,9]. However, unsupported cobalt oxides exhibit relatively low electrical conductivity, particle agglomeration, structural reconstruction, or partial dissolution during prolonged polarization [10,11]. Immobilizing cobalt species on a stable oxide support or incorporating Co into a host lattice may improve their dispersion, modify their electronic structure, and enhance charge transfer and structural stability. Titanium oxides are particularly attractive host materials because of their abundance, chemical robustness, corrosion resistance, and versatile defect chemistry [12,13].

Co-containing titanium oxides have been studied in different structural forms, including Co-doped TiO_2_, cobalt oxide-decorated TiO_2_, mixed Co–Ti oxides, and cobalt titanates [14,15,16]. Depending on the synthesis conditions and cobalt content, Co may substitute for Ti within the oxide lattice, occupy interstitial or defect-associated sites, or form highly dispersed CoO_x_ surface domains. These configurations provide a variety of electronic structures and electrocatalytic properties. Seitz et al. found that the OER activity of CoTiOx catalysts is highly dependent on the Co/Ti ratio and electronic state, with higher cobalt content (not less than 1:1) promoting active Co^3+^ species, whereas high titanium content stabilizes less active Co^2+^. Additionally, lower-temperature calcination maintains an amorphous structure, which is more effective for OER than the phase-segregated, crystalline states formed at higher temperatures [17]. Thus, the role of titanium is not limited to physically supporting the Co species but also includes modification of their local and electronic structures.

Cobalt incorporation into TiO_2_ can also promote the formation and stabilization of oxygen vacancies, increase charge-carrier density, and suppress oxidative surface passivation [18,19,20]. Yang et al. demonstrate that cobalt-doped black TiO_2_ nanotube arrays enhance electrocatalytic activity and stability by creating surficial oxygen vacancies that prevent passivation and by improving electrical conductivity [20]. Similarly, mesoporous TiO_2_ decorated with small amounts of cobalt oxide showed considerably improved OER activity compared with pristine TiO_2_ because of the uniform dispersion of CoO_x_ species, increased accessibility of active sites, and favorable CoO_x_–TiO_2_ interfacial interactions [21]. Co_3_O_4_-modified TiO_2_ composites have also exhibited activity toward both the OER and ORR in alkaline media, demonstrating the potential of Co–Ti–O interfaces for bifunctional oxygen electrocatalysis [22]. However, the low intrinsic electrical conductivity of stoichiometric TiO_2_ remains an important limitation, particularly when TiO_2_ constitutes a substantial fraction of the electrode material [23].

This limitation may be overcome by partially reducing TiO_2_ and forming oxygen-deficient titanium oxides. Among these compounds, Magnéli phases (MP) represent a homologous series with the general formula Ti_n_O_2n−1_, commonly with n being between 3 and 10 [24,25,26,27]. Their crystal structures are derived from rutile TiO_2_ through the periodic introduction of crystallographic shear planes, accompanied by the presence of mixed Ti^3+^ and Ti^4+^ oxidation states [28,29]. This structural and electronic arrangement gives MP markedly higher electrical conductivity than stoichiometric TiO_2_ while preserving high chemical, thermal, and electrochemical stability [30,31]. These properties have led to their application as electrodes and conductive catalyst supports in fuel cells, batteries, water electrolysis, electrosynthesis, and electrochemical water treatment [12,26,28].

Among the MPs, Ti_4_O_7_ has attracted the greatest attention because of its particularly high electrical conductivity and chemical resistance [12,26,28,32]. Ti_4_O_7_ has been examined as both an electrocatalyst and a conductive support for noble-metal and transition-metal active phases. Li et al. demonstrated that Ti_4_O_7_-based electrodes can catalyze the ORR and possess characteristics relevant to rechargeable metal–air batteries [32]. Ti_4_O_7_ has also been investigated as a support for Pt and Pt–Ir nanoparticles, where strong catalyst–support interactions and the corrosion resistance of the MP contributed to improved ORR and OER performance [32,33]. Furthermore, Ti_4_O_7_-based materials have been explored as stable oxygen electrodes and bipolar-plate materials because of their low electrical resistance and resistance to aggressive electrochemical environments [34,35]. Other MPs, such as Ti_5_O_9_, Ti_6_O_11_, and Ti_8_O_15_, are particularly interesting because they occupy an intermediate compositional and electronic position between highly reduced Ti_4_O_7_ and stoichiometric TiO_2_. Their mixed-valence Ti^3+^/Ti^4+^ structure provides appreciable electronic conductivity, whereas higher oxygen content and different periodicity of crystallographic shear planes may influence surface adsorption, oxygen-vacancy distribution, and the activation of oxygen-containing intermediates [36,37]. The reported electrical conductivities of Ti_5_O_9_, Ti_6_O_11_, and Ti_8_O_15_ are lower than those of Ti_4_O_7_ but remain several orders of magnitude higher than that of stoichiometric TiO_2_, making it a potentially attractive electrocatalyst or catalyst support [12]. However, most reported applications of TSO have been related to electronic materials, conductive ceramics, environmental electrooxidation, or catalyst-support applications rather than oxygen electrocatalysis [36]. For example, Au/Ti_6_O_11_ heterostructures have been investigated for the hydrogen evolution reaction, demonstrating that Ti_6_O_11_ can participate in favorable interfacial electronic interactions with deposited catalytic species [38].

Doping and surface modification enable tailoring the catalytic properties of MP titanium oxides. The incorporation of transition metals changes the Ti^3+^/Ti^4+^ ratio, oxygen-vacancy concentration, electrical conductivity, adsorption energies of reaction intermediates, and resistance to surface oxidation [28,39]. Transition-metal species located at the surface or incorporated into the Magnéli structure simultaneously introduce new catalytic centers and promote electronic interactions with the conductive titanium suboxide framework. Nevertheless, most studies of modified MP have focused on Ti_4_O_7_, while Ti_5_O_9_, Ti_6_O_11_, and other higher members of the series remain considerably less explored [39,40].

The potential of combining cobalt with MP titanium suboxides has previously been demonstrated using commercial Ebonex as a conductive catalyst support. Paunović et al. deposited metallic Co onto mechanically treated Ebonex by a sol–gel approach and evaluated the resulting Co/Magnéli electrocatalysts toward hydrogen and oxygen evolution. The catalytic activity increased with decreasing particle size of the MP support, while the Co/Ebonex system exhibited particularly promising behavior toward the OER, highlighting the potential of interactions between cobalt-based active sites and conductive MP supports [41]. Among the less-explored MPs, Ti_5_O_9_ has been reported to exhibit high electronic conductivity together with high chemical and thermal inertness, supporting its potential as an electrochemically functional electrode material [42]. Oxygen reduction has also been investigated on a mixed Magnéli-phase ceramic consisting predominantly of Ti_5_O_9_, demonstrating ORR activity of Ti_5_O_9_-rich titanium suboxide materials [43]. Ti_8_O_15_ is particularly attractive from the perspective of catalyst-support design. Single-crystalline Ti_8_O_15_ nanowires have been employed as conductive carbon-free supports for Pt nanoparticles, with Pt/Ti_8_O_15_ showing enhanced activity and durability for methanol electrooxidation compared with commercial Pt/C [44]. The same support concept was subsequently demonstrated for Pd nanoparticles, where Pd/ Ti_8_O_15_ exhibited enhanced ethanol oxidation activity and improved durability relative to Pd/C [45].

Introducing Co into MPs is a promising route for combining the redox activity of Co centers with the conductivity/chemical stability of an MP framework. Co incorporation may alter the Ti^3+^/Ti^4+^ distribution, generate or stabilize oxygen vacancies, and modify the electronic density of neighboring titanium and oxygen atoms. Alternatively, highly dispersed Co species on the MP or rutile surface may form Co–O–Ti interfacial sites with favorable binding strengths for OH*, O*, OOH*, and O_2_* intermediates. The conductive titanium (sub)oxide matrix could facilitate electron transport between these sites and the electrode while suppressing the agglomeration of cobalt species. Such effects could be beneficial for both the OER and ORR, although an optimal balance is required because excessively strong or weak adsorption of oxygen intermediates is unfavorable for overall catalytic kinetics [46].

Despite this potential, MPs have not been systematically investigated as OER and ORR electrocatalysts, and the influence of cobalt modification on their bifunctional oxygen activity remains unknown. Following our contributions on MP in HER and OER activity [36] as well as electrooxidation of pollutants [37], in this work, Co-modification of MPs was comprehensively characterized. The relationship between cobalt incorporation, structural and surface properties, and electrochemical behavior was established. The activity and kinetics toward the OER and ORR and contributions of cobalt sites and mixed titanium phases were investigated. Exploring the electrocatalytic properties of the materials obtained by Co-modification of MPs expands their application as non-precious bifunctional oxygen electrocatalysts.

## 2. Materials and Methods

### 2.1. Material Preparation

Titanium dioxide powder (TiO_2_, 99.5%; Evonik Industries AG, Essen, Germany), cobalt(II) nitrate hexahydrate (Co(NO_3_)_2_·6H_2_O; Sigma-Aldrich, St. Louis, MO, USA), urea (CO(NH_2_)_2_; Sigma-Aldrich, St. Louis, MO, USA), and Nafion solution (5 wt%, Sigma-Aldrich, St. Louis, MO, USA) were used as received. Ultrapure water was used for the preparation of all aqueous solutions.

Titanium suboxide (TSO) was prepared from TiO_2_ powder by thermal treatment at 950 °C for 8 h under a reducing atmosphere containing 5% H_2_ in Ar, slightly modified from [37]. Co-modification of TSO was performed by hydrothermal deposition. Briefly, 0.04 mmol of Co(NO_3_)_2_·6H_2_O and 0.20 mmol of urea were dissolved in 50 mL of distilled water, after which 75 mg of TSO powder was added. The suspension was transferred into a Teflon-lined stainless-steel autoclave and maintained at 180 °C for 5 h. After naturally cooling to room temperature, the solid product was washed and dried before being subjected to another treatment at 950 °C for 12 h under a reducing atmosphere to obtain Co-modified material, denoted as Co/TO.

### 2.2. Characterization

Structural analysis of the synthesized powders was conducted using a Rigaku Ultima IV diffractometer (Rigaku, Tokyo, Japan) operating in the Bragg–Brentano configuration. XRPD patterns were collected using Cu Kα radiation (λ = 1.54178 Å) over a 2θ range from 10° to 80°, with a step size of 0.050° and a scanning speed of 1° min^−1^.

Fourier-transform infrared (FTIR) measurements were performed on a Nicolet iS20 spectrometer (Thermo Fisher Scientific, Waltham, MA, USA) using the KBr pellet method. The spectra were acquired over the 4000–400 cm^−1^ wavenumber range at a spectral resolution of 2 cm^−1^, with 32 scans collected for each measurement.

Raman measurements were carried out using a DXR Raman Microscope (Thermo Fisher Scientific, Waltham, MA, USA) equipped with a 532 nm diode-pumped solid-state laser. The laser was focused onto the sample surface to a spot size of 2.1 μm using a 10× microscope objective, with the laser power at the sample maintained at 2 mW. Each spectrum was acquired with a 10 s exposure time and 10 accumulations. The scattered radiation was collected and analyzed using a spectrograph fitted with a 50 μm pinhole and a 900 lines mm^−1^ diffraction grating. For each sample, spectra were recorded at five randomly selected positions, and the spectrum presented herein represents their average.

A Phenom ProX scanning electron microscope (Thermo Fisher Scientific, Waltham, MA, USA) was used to obtain surface images coupled with energy-dispersive X-ray spectroscopy (EDX) to confirm Co surface modification. Imaging was performed at magnifications of 10,000× and 20,000× using a backscattered electron detector (BSD Full) operated at 15 kV in Map mode.

### 2.3. Electrochemical Measurements

Electrochemical measurements were performed using a glassy-carbon (GC) electrode modified with a thin film of TSO or Co/TO. The catalyst ink was prepared by dispersing 5 mg of the investigated material in 0.5 mL of an ethanol/water/Nafion mixture, followed by ultrasonication until a homogeneous suspension was obtained. Subsequently, 10 μL of the ink was drop-cast onto a GC disk electrode with a diameter of 5 mm and allowed to dry at room temperature. The resulting catalyst loading was approximately 250 μg cm^−2^.

The ORR activity was initially evaluated by cyclic voltammetry using a conventional three-electrode electrochemical cell. The catalyst-modified GC electrode, carbon rod, and saturated calomel electrode (SCE) were used as the working, counter, and reference electrodes, respectively. An aqueous 1 M KOH solution was used as the supporting electrolyte. All measurements were performed using an Ivium VO1107 potentiostat/galvanostat (Ivium Technologies B.V., Eindhoven, The Netherlands) under ambient conditions at atmospheric pressure and room temperature. Before and throughout the ORR measurements, the electrolyte was saturated with O_2_ using a continuous gentle oxygen flow. Background voltammograms were recorded in N_2_-saturated electrolyte to eliminate contributions unrelated to oxygen reduction.

Peroxide formation was further investigated using a rotating ring–disk electrode (RRDE) comprising a GC disk and a Pt ring. The catalyst-coated GC disk served as the working electrode, while the Pt ring detected HO_2_^−^ generated during oxygen reduction at the disk. The geometric areas of the disk and ring were 0.19625 and 0.1709 cm^2^, respectively, and the RRDE collection efficiency was (N = 0.36). Measurements were performed in O_2_-saturated 1 M KOH at rotation rates of 400, 900, 1600, 2500, and 3600 rpm. The disk and ring current densities were normalized to their respective geometric surface areas.

The HO_2_^−^ yield was calculated from the simultaneously measured disk and ring currents using Equation (1):

(1) %HO_2_^−^ = 2(I_ring_/N)/(I_disc_ + I_ring_/N)

where *I_ring_* is the ring current, *I_disc_* is the disc current, and *N* is the collection efficiency.

The average electron-transfer number per O_2_ molecule was calculated using Equation (2):

(2) n = 4I_disc_/(I_disc_ + I_ring_/N)

The OER investigation of TSO and Co/TO was performed by linear sweep voltammetry (LSV) at 1600 rpm and 20 mV s^−1^. Electrochemical impedance spectroscopy (EIS) measurements were performed in 1 M KOH using the same three-electrode configuration in the 0.1–100,000 Hz range at 1.7 V. The impedance spectra were fitted using the Randles equivalent circuit, where R_e_ represents the uncompensated series resistance, R_ct_ corresponds to the interfacial charge-transfer resistance, and the constant-phase element accounts for the non-ideal capacitive response of the heterogeneous catalyst layer. All presented potentials were converted to the reversible hydrogen electrode (RHE).

## 3. Results and Discussion

### 3.1. Materials Characterization

X-ray diffraction analysis of pristine TSO confirmed the formation of titanium suboxide MP, with the diffraction pattern most consistently described by a combination of Ti_5_O_9_ and Ti_8_O_15_ (Figure 1).

The reflections at 2θ = 26.4°, 35.5°, and 37.5° could be assigned to Ti_5_O_9_ (PDF# 00-051-0641), whereas those at approximately 26.7°, 28.1°, and 63.3° belong to Ti_8_O_15_ (PDF# 00-050-0790). In addition to the MP, the diffractogram contains characteristic reflections of rutile at approximately 2θ = 27.6°, 36.3°, 39.4°, 41.4°, 44.2°, 54.5°, 56.7°, 64.2°, and 69.2°, indicating that a fraction of TiO_2_ remained unreduced under the applied synthesis conditions. The presence of the rutile TiO_2_ phase is clearly evident in the Raman spectra shown in the upper row of Figure 2, with characteristic strong Raman bands at 606 and 445 cm^−1^, a medium-intensity band at 240 cm^−1^, and a very weak band at 149 cm^−1^ [47], Figure 2. Although MPs were identified by XRD, their characteristic Raman features could not be unambiguously resolved in the spectra. This may be related to the structural disorder of the MP, as well as to the strong Raman response of the TiO_2_ phase, which can dominate the spectrum and obscure weaker features arising from TSO.

The reduction of the pristine sample was intentionally stopped at this intermediate stage (i.e., 8 h at 950 °C, 5% H_2_ in Ar), characterized by the coexistence of rutile TiO_2_ and emerging MP. This state was selected as a reference point to test the hypothesis that the presence of transition metals on the surface could promote further transformation to lower titanium suboxides, with possible TSO doping, or strong Co interaction with TSO. However, with cobalt addition, the XRD pattern showed reflections corresponding to rutile TiO_2_ and metallic fcc Co. At the same time, characteristic Co_3_O_4_ features are observed in the Raman spectra at 694 and 522 cm^−1^ [48] (upper row of Figure 2b). Additionally, weaker features of Co–O vibrations are also discernible in the FTIR spectra at about 630 and 560 cm^−1^ (lower row of Figure 2b). These findings indicate that cobalt oxide species remained to some extent in the sample after the treatment and/or may have formed subsequently upon exposure to atmospheric conditions. Since fcc Co represents the high-temperature polymorph of metallic cobalt, its presence suggests that cobalt oxide species formed under hydrothermal treatment underwent reduction to the metallic state under the applied conditions.

A profile matching procedure was applied to the Co/TO diffractogram with the aim of calculating unit cell parameters for the rutile phase. They were found to be a = 4.595(2) Å and c = 2.959(3) Å. Comparing with literature values a = 4.954 Å and c = 2.958 Å [49], it is clear that no significant changes in unit cell parameters can be detected. At the same time, no clear enhancement of the rutile-to-Magnéli transformation was observed, indicating that the initial hypothesis of Co-assisted acceleration of this transformation was not supported under these conditions. Instead, Co stayed in the metastable phase on the grain surface.

Comparable behavior reported for other transition metals further demonstrates the strong dopant dependence of this process. Ni/TiO_2_ systems under reducing conditions may retain a reduced rutile-like structure together with metallic Ni [50], while Fe can either substitute into the rutile lattice or segregate into Fe-containing oxide phases at higher concentrations [51]. Likewise, Mn has been shown to affect the anatase-to-rutile transformation without necessarily promoting MP formation [52]. In Cr-doped rutile, low Cr concentrations yield rutile solid solutions without detectable crystallographic shear planes, while ordered Cr-containing Magnéli structures occur only at substantially higher dopant contents [53]. These observations indicate that oxygen-vacancy generation alone is insufficient to guarantee the formation or stabilization of MP; the critical step is the ordering of these defects into crystallographic shear structures.

Scanning electron microscopy coupled with EDX was used to confirm the deposition as well as atomic content on the surface (Figure 3, Table 1). SEM images of pristine TSO resemble characteristic elongated grain structures typical of MP [54], forming a continuous, porous network, while the Co/TO sample showed identical titanium support morphology with spherical cobalt nanoparticles on top. EDX showed cobalt content of around 4 at.% with homogeneous dispersion of nanoparticles. The higher Co content detected by EDX compared with the nominal Co loading is attributed to the preferential deposition and distribution of Co on the TSO particle surfaces, and therefore represents a local surface-enriched composition rather than the bulk Co loading.

### 3.2. Oxygen Reduction and Peroxide Formation on TSO and Co/TO Samples

TSO and Co/TO were examined by cyclic voltammetry, recorded in the potential range corresponding to the non-Faradaic region and in the scan rate region from 10 to 50 mV s^−1^ (Figure S1). The obtained cyclic voltammograms (CVs) were used for estimation of double-layer capacitance (C_dl_) values, which are proportional to the electrochemical active surface area (ECSA). C_dl_ values of 15 and 39 µF cm^−2^ were obtained for TSO and Co/TO, respectively. It is evident that Co/TO gives C_dl_ values almost 2.6 times higher than the TSO sample, indicating higher surface availability to the reactants.

Figure S2 shows no reduction peak in the CVs of TSO and Co/TO in the N_2_-saturated electrolyte, while well-defined ORR peaks were observed in O_2_-saturated solution at 0.63 and 0.61 V for TSO and Co/TO, respectively. The obtained ORR peak potentials are in agreement with a similar literature report [55]. These results showed high ORR activity of the synthesized samples, where, e.g., the peak current density of Co/TO increased from −0.0035 in N_2_-saturated to −0.2747 mA cm^−2^ in O_2_-saturated solution (Figure S2).

The onset potential (E_onset_) observed at a current density of −0.1 mA cm^−2^ and the corresponding half-wave potential (E_1/2_) were obtained from LSV RDE curves (Figure 4a) for both samples (Figure 4) in O_2_-saturated solution. Namely, E_onset_ values of 0.73 and 0.72 V and corresponding E_1/2_ values of 0.63 and 0.61 V were observed for TSO and Co/TO, respectively. These values are almost the same as or similar to those reported in the literature [56,57,58]. Namely, E_1/2_ of 0.73 V was obtained for the cation-ordered perovskite oxide originating from Ba_0.5_Sr_0.5_Co_0.8_Fe_0.2_O_3−δ_ electrodes, and 0.72 and 0.63 V for E_onset_ and E_1/2_ were obtained for iron–copper-doped reduced graphene oxide (FeCu/rGO) [56,57], which are somewhat higher or similar to the values obtained here. Cobalt–iron supported on carbon aerogel (Co-Fe/CA-2) gave 0.77 and 0.63 V for E_onset_ and E_1/2_ [58], similar to the values obtained here for Co/TO electrocatalysts during ORR.

Tafel slopes of 99 and 112 mV dec^−1^ were calculated for synthesized TSO and Co/TO (Table 2). Porous NiO/CoN interface nanowire arrays (NiO/CoN PINWs) gave a Tafel slope of 138 mV dec^−1^ [59], and CoFe-PBA/C450 gave 136.2 mV dec^−1^ [58], which is significantly higher than the Tafel slope measured here, indicating admirable ORR kinetics on Co/TO with respect to similar material types. The rest of the electrocatalysts presented in Table 2 showed comparable or somewhat higher Tafel slopes than those obtained here. Short-term stability was probed by chronoamperometric measurement for Co/TO (Figure 4d) and showed stable current during a one-hour test after the initial 70% drop.

LSV RDE results obtained at different scan rates are presented in Figure 5 and were used to obtain the number of electrons exchanged (n) during ORR by the Koutecky–Levich equation [61]. Namely, n ranges from 2.51 to 2.54 and from 2.94 to 3.00 were calculated for TSO and Co/TO (Table 2), which is in agreement with similar literature reports [57,58,60]. For both materials, the cathodic disk current began to increase at approximately −0.1 to −2.5 mA cm^−2^ and became strongly dependent on the rotation rate at more negative potentials.

The increase in current density with rotation rate confirms that oxygen transport from the bulk electrolyte to the electrode surface significantly contributes to the overall reaction rate. However, neither material exhibited a clearly defined diffusion-limited plateau within the investigated potential range. Instead, the current continued to increase toward more negative potentials, indicating mixed kinetic–mass-transport control. Such behavior can also be associated with the porous and heterogeneous catalyst coatings, within which oxygen transport, electrolyte penetration, and electron transfer occur over a distribution of reaction sites. At 0.3 V, the cathodic current density of pristine TSO increased from approximately −1 mA cm^−2^ at 400 rpm to −2.5 mA cm^−2^ at 3600 rpm. Comparable values were obtained for Co/TO, ranging from approximately −1 to −2.25 mA cm^−2^ over the same rotation-rate interval. Thus, cobalt modification did not substantially shift the ORR onset potential or increase the measured current density under strong mass-transport control. At the highest rotation rates, the pristine material even exhibited a slightly higher absolute disk current. The principal effect of cobalt modification is not simply an increase in total ORR current but a change in interfacial charge-transfer properties and product distribution.

Additionally, the oxygen reduction reaction on pristine TSO and Co/TO was investigated using rotating ring–disk electrode measurements at rotation rates between 400 and 3600 rpm (Figure 5). A positive ring current appeared concurrently with the disk ORR current for both electrodes. Under the selected ring potential, this signal is attributed to the oxidation of H_2_O_2_/HO_2_^−^ generated at the disk. The ring response confirms that peroxide formation is a major oxygen-reduction pathway on both TO-based materials. For pristine TSO, the ring current density at 0.3 V increased from approximately 0.35 mA cm^−2^ at 400 rpm to 0.56 mA cm^−2^ at 3600 rpm. The corresponding ring current densities for Co/TO were slightly lower, increasing from approximately 0.33 to 0.48 mA cm^−2^. The lower ring currents of Co/TO, despite comparable disk currents, indicate that a smaller fraction of the reduced oxygen leaves the disk as detectable peroxide. Cobalt modification moderately promotes more complete oxygen reduction and/or the subsequent reduction of initially formed peroxide. This conclusion is supported by the electron-transfer numbers calculated from the RRDE data. For pristine TSO, *n* increased from 2.14 at 400 rpm to 2.52 at 3600 rpm, whereas Co/TO provides values ranging from 2.24 to 2.60. The values remain much closer to 2 than to 4, confirming that the 2-electron ORR pathway leading to H_2_O_2_/HO_2_^−^ predominates on both electrodes. However, values above two demonstrate a simultaneous contribution from the four-electron pathway to water or hydroxide and/or further electroreduction of the peroxide intermediate. The apparent peroxide yield of pristine TSO decreased from approximately 93.1% at 400 rpm to 73.9% at 3600 rpm. For Co/TO, it decreased from approximately 88.2% to 69.9%. The calculated values are summarized in Table 3.

The consistently higher *n* values and lower peroxide yields of Co/TO show that cobalt modification shifts the product distribution slightly toward the four-electron pathway. Li et al. reported electron-transfer numbers of 2.3–2.9 and H_2_O_2_ yields of 35–72% for Ti_4_O_7_ in 1–6 M KOH, indicating parallel 2- and 4-electron pathways [32]. Here we record a moderately lower *n*, as Co-containing surface sites may bind oxygenated intermediates more strongly, facilitate O–O bond cleavage, or promote the secondary reduction of H_2_O_2_/HO_2_^−^, as Safeen et al. have shown [62]. Interfacial Co–O–Ti sites may also alter the electronic density of neighboring titanium and oxygen atoms and thereby change the adsorption energies of O_2_*, OOH*, O*, and OH*, as shown by Bharti and Modak [63]. Since *n* remains below 2.61, this demonstrates that peroxide formation remains the dominant pathway after cobalt modification. Pristine TSO is slightly more selective for peroxide production, whereas Co/TO provides a modestly greater contribution from more complete oxygen reduction. For both materials, *n* increased with rotation rate. Since the intrinsic electron-transfer number would ideally be independent of hydrodynamic conditions, this trend should be interpreted cautiously. It may result from mixed kinetic–mass-transport control, the porous nature of the catalyst layer, differences in peroxide readsorption and secondary reduction, or deviations in the effective collection efficiency caused by the finite thickness and non-uniformity of the deposited film [64,65]. TSO retained the higher peroxide selectivity at every investigated rotation rate. This agrees with reports that Co_3_O_4_–TiO_2_ interfaces enhance oxygen electrocatalysis through increased charge-carrier density and favorable electronic interactions [22]. Moreover, selectivity of Co-based catalysts is found to be strongly dependent on the coordination environment and support, while suitably configured Co sites can retain high activity for two-electron oxygen reduction to H_2_O_2_ [66].

Both TSO and Co/TO predominantly catalyze the two-electron reduction of oxygen and consequently show considerable potential for electrosynthetic peroxide production. Pristine TSO provides the higher peroxide selectivity, reaching approximately 93% under the investigated conditions, whereas Co/TO exhibits markedly lower interfacial resistance and a modest shift toward the four-electron pathway. Thus, cobalt modification offers an effective means of tuning the balance between charge-transfer kinetics and peroxide selectivity in TSO-based oxygen-reduction catalysts. Overall, cobalt modification only slightly affects the ORR activity, while altering the balance between peroxide selectivity and the contribution of the four-electron pathway.

### 3.3. Oxygen Evolution Reaction on TSO and Co/TO

The polarization curves demonstrate that pristine TSO is essentially inactive toward the OER within the investigated potential range, whereas Co modification produces a pronounced anodic response above approximately 1.5 V vs. RHE (Figure 6). Using 1 mA cm^−2^ as an operational onset criterion, Co/TO reaches this current density at approximately 1.55 V vs. RHE, corresponding to an onset overpotential of about 350 mV. A current density of 10 mA cm^−2^ is achieved at approximately 1.83 V vs. RHE, giving η_10_ ≈600 mV. These values show that cobalt introduces catalytically active sites that are absent from pristine TSO, although the activity remains below that of optimized high-surface-area Co–Ti oxide catalysts. For example, Co-modified mesoporous TiO_2_ showed an onset potential of approximately 1.55 V at 1 mA cm^−2^ and an overpotential of about 350 mV at higher current densities [21]. Co_3_O_4_-modified low-symmetry mesoporous TiO_2_ and CoO_x_/Co-doped black TiO_2_ nanotubes required only 348 and 352 mV, respectively, to reach 10 mA cm^−2^ [20,22]. Their superior performance is likely related to higher surface areas, larger Co loadings, and directly exposed CoO_x_/CoOOH active domains. In contrast, the present material contains a low nominal Co content within or on the relatively low-surface-area TSO matrix. On the other hand, zinc and iron copper nanoparticles deposited on reduced graphene oxide (ZnCu/rGO [67] and FeCu/rGO [57]) showed E_onset_ values of 1.66 and 1.64 V, which are the same as the value obtained for TMS and 110 mV more positive than the value obtained for Co/TO (Table 4). IrO_2_ gives an E_onset_ of 1.52 V [67], which is almost the same as the Eonset obtained here for Co/TO (Table 4). Metal oxides based on Mn_2_O_3_ with TiO_2_ were synthesized, and Pt or PtNi nanoparticles were anchored on them (PtNi/Mn_2_O_3_- TiO_2_) and examined for OER in alkaline media [68], where it showed an E_onset_ of 1.73 V, which is 180 mV more positive than the value obtained here for Co/TO.

To further investigate the OER kinetics of the prepared catalysts, Tafel analysis was performed by plotting the potential values against the logarithm of the current density. The obtained Tafel slopes were 327 mV dec^−1^ for TSO and 56 mV dec^−1^ for Co/TO, indicating substantially more favorable OER reaction kinetics for the Co-modified sample (Figure 6b). The considerably lower Tafel slope of Co/TO suggests that the incorporation of Co into the TSO structure facilitates faster electron-transfer processes and promotes more favorable reaction kinetics compared to the pristine TSO catalyst. For comparison, the obtained Tafel slopes were evaluated against those reported for several Zn- and Co-based OER electrocatalysts. The Tafel slope of 101 mV dec^−1^ reported for ZnCu/rGO [67] is notably higher than that of Co/TO, while FeCu/rGO exhibits an even larger value of 149 mV dec^−1^ [57]. A similar Tafel slope of 101 mV dec^−1^ was reported for CoPt/rGO [69], whereas PtNi/Mn_2_O_3_-TiO_2_ shows a considerably higher value of 224 mV dec^−1^ [68]. Among the Zn-containing catalysts, ZnCo/(Ppy/CNTs) (Ppy/CNTs—polypyrrole/carbon nanotubes) exhibits a Tafel slope of 228.1 mV dec^−1^, while Co-Zn ZIF (ZIF-zeolitic imidazolate frameworks) catalysts show values of 100 and 73 mV dec^−1^ for samples prepared for 2 and 24 h, respectively. Co(Zn)S_2_/CC (CC—carbon cloth) demonstrates a Tafel slope of 72.9 mV dec^−1^, indicating favorable OER kinetics [70]. Overall, the Tafel analysis confirms that Co incorporation has a pronounced positive effect on the OER kinetics of TSO. The Co/TO catalyst combines an onset potential of 1.55 V, an onset overpotential of 350 mV, and a Tafel slope of 56 mV dec^−1^, demonstrating competitive OER characteristics compared with a range of previously reported Zn- and Co-based electrocatalysts. In particular, its Tafel slope is comparable to or lower than those reported for several highly active transition-metal-based OER catalysts, highlighting Co/TO as a promising candidate for efficient oxygen evolution.

The substantial enhancement relative to pristine TSO is substantiated by the impedance results, which showed a decrease in charge-transfer resistance from 17,450 to 47.4 Ω following Co modification (Figure 6c). R_ct_ decreased by a factor of approximately 368, corresponding to a reduction of about 99.7%. The substantially smaller semicircle observed for Co/TO shows that cobalt modification facilitates interfacial charge transfer. A similar improvement following Co incorporation has been reported for Co-doped black TiO_2_, where cobalt generated and stabilized surface oxygen vacancies, increased charge transport, and limited surface passivation [20]. Here, the reported R_ct_ is slightly lower than reported for the carbon-supported Ti_4_O_7_/CNT catalyst, despite the absence of a highly conductive carbon network [71].

**Table 4:** Comparison of OER kinetic parameters of TSO and Co/TO electrocatalysts with similar cobalt- or transition metal-based literature reports on electrocatalysts.

OER Electrocatalysts	E_onset_/V	η_onset_/mV	b/mV dec^−1^	References
TSO	1.69	460	327	This work
Co/TO	1.55	350	56	This work
ZnCu/rGO	1.66	510	101	[67]
FeCu/rGO	1.64	607	149	[57]
IrO_2_	1.52	555	115	[67]
CoPt/rGO	1.61	410	101	[69]
PtNi/Mn_2_O_3_-TiO_2_	1.73	530	224	[68]
ZnCo/(Ppy/CNTs)_4_	*/*	/	228.1	[72]
Co-Zn ZIF(2h)	*/*	/	100	[73]
Co-Zn ZIF(24h)	*/*	/	73	[73]
Co(Zn)S_2_/CC	/	/	72.9	[70]

The very high R_ct_ of pristine TSO suggests that the electrochemical response is strongly restricted by the properties of the actual electrode interface. Possible contributions include partial surface oxidation, insufficient electrical contact between TSO particles, and limited electrolyte accessibility within the coating. The significant decrease in R_ct_ following Co modification suggests the introduction of electrochemically accessible surface sites and improved electronic communication between the TO framework and adsorbed oxygen intermediates. Short-term stability of Co/TO towards OER was probed by chronoamperometric measurement at 1.7V (Figure 6d) and showed current during the 20-min test.

The increase in double-layer capacitance from 15 to 39 μF cm^−2^ suggests a larger electrochemically accessible surface area and a higher number of accessible surface sites after Co modification. The simultaneous decrease in charge-transfer resistance indicates more favorable interfacial charge-transfer kinetics, which is consistent with the pronounced enhancement of OER activity observed for Co/TO. Previous studies have reported that Co-containing oxide and oxyhydroxide species can provide active sites for OER in alkaline media, where Co-OH and Co-O-containing species participate in the formation of OER intermediates [74,75]. Moreover, catalytic activity depends on both the number and accessibility of active sites and their interaction with reaction intermediates [74,76]. In the present system, these changes in surface accessibility and interfacial charge transfer may therefore contribute to the enhanced OER activity, whereas the relatively small changes in ORR activity suggest a more limited effect of Co modification on the overall ORR performance.

## 4. Conclusions

Herein, cobalt was deposited on titanium suboxide under mild conditions. Contrary to expectations, Co decoration led to a phase reversal, resulting in Co being deposited on the rutile TiO_2_ surface. Suspected unshearing of crystallite planes and reversion to rutile TiO_2_ could be related to Co reacting with oxygen vacancies between rutile grains, which form TSO, but further XRD/HR-TEM evidence is needed. In this way, Co served as a catalyst for phase reversal and not as a potential dopant, as initially intended. Low-level surface content (4.15 wt.%) was confirmed by SEM-EDX measurements, confirming homogeneous dispersion of cobalt on the surface. Raman and FTIR measurements confirmed changes in the surface state of TSO and the presence of Co. Decoration of Co on TSO significantly improved OER, with charge transfer resistance for OER diminishing from 17.5 kΩ to 47.4 Ω, indicating excellent activity towards oxygen evolution, with onset potential for OER shifting to 1.55 V vs. RHE. Co surface deposition only slightly affected the oxygen reduction reaction and peroxide production of TSO, while both TSO and Co/TO exhibited clear ORR activity under the investigated conditions.

## Figures and Tables

**Figure 1:**
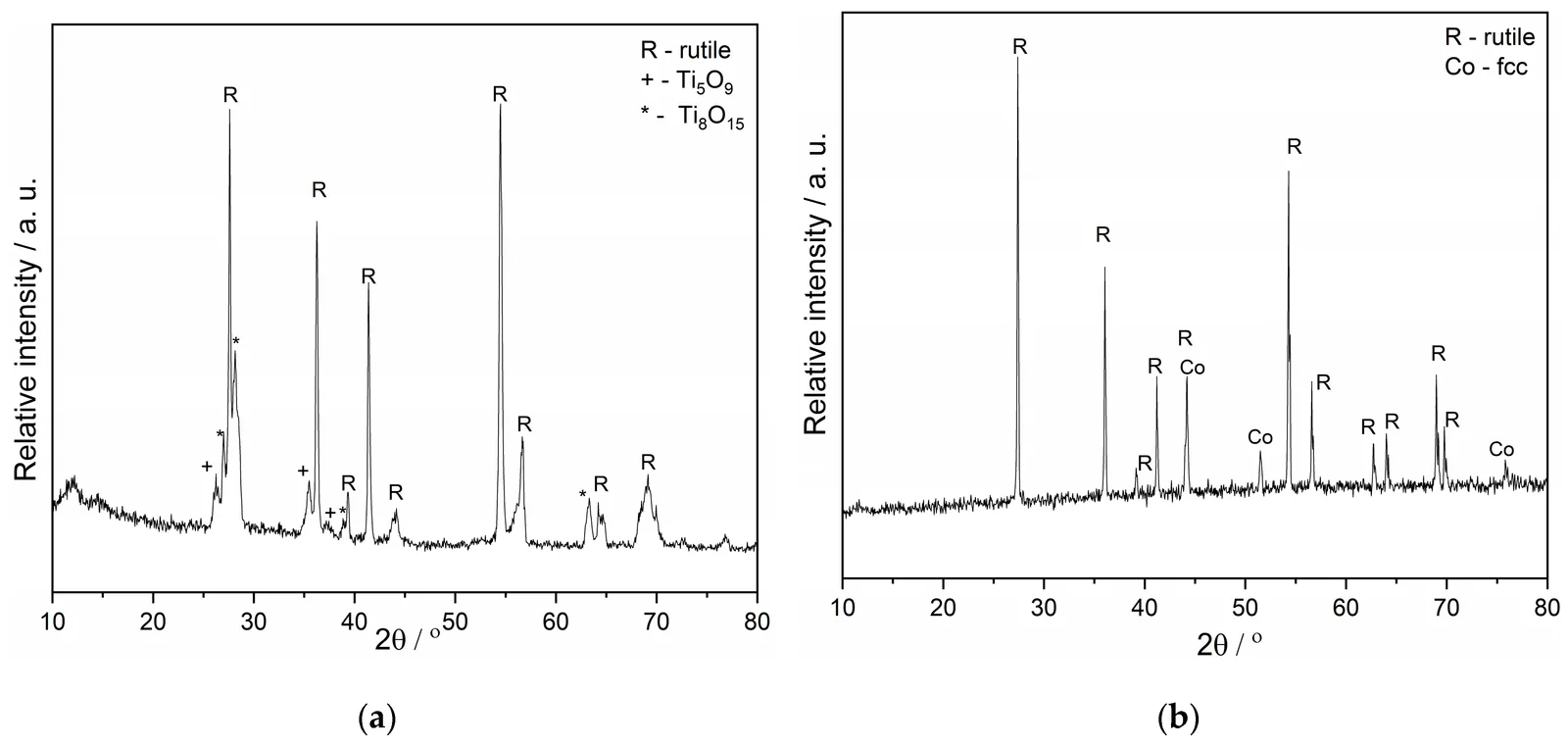
Diffractograms of (**a**) pristine TSO and (**b**) Co/TO sample.

**Figure 2:**
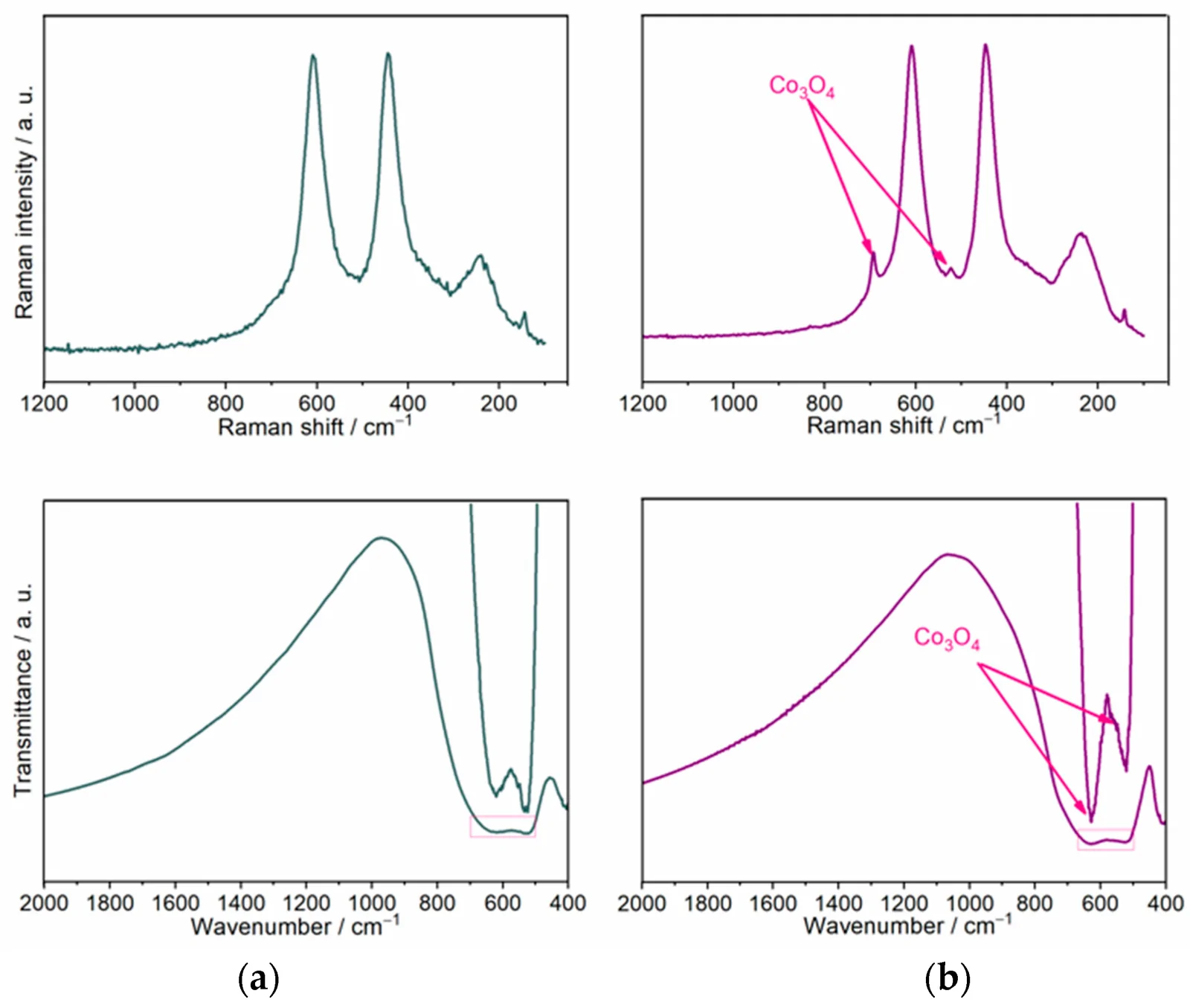
Raman and FTIR spectra of (**a**) pristine TSO and (**b**) Co/TO sample.

**Figure 3:**
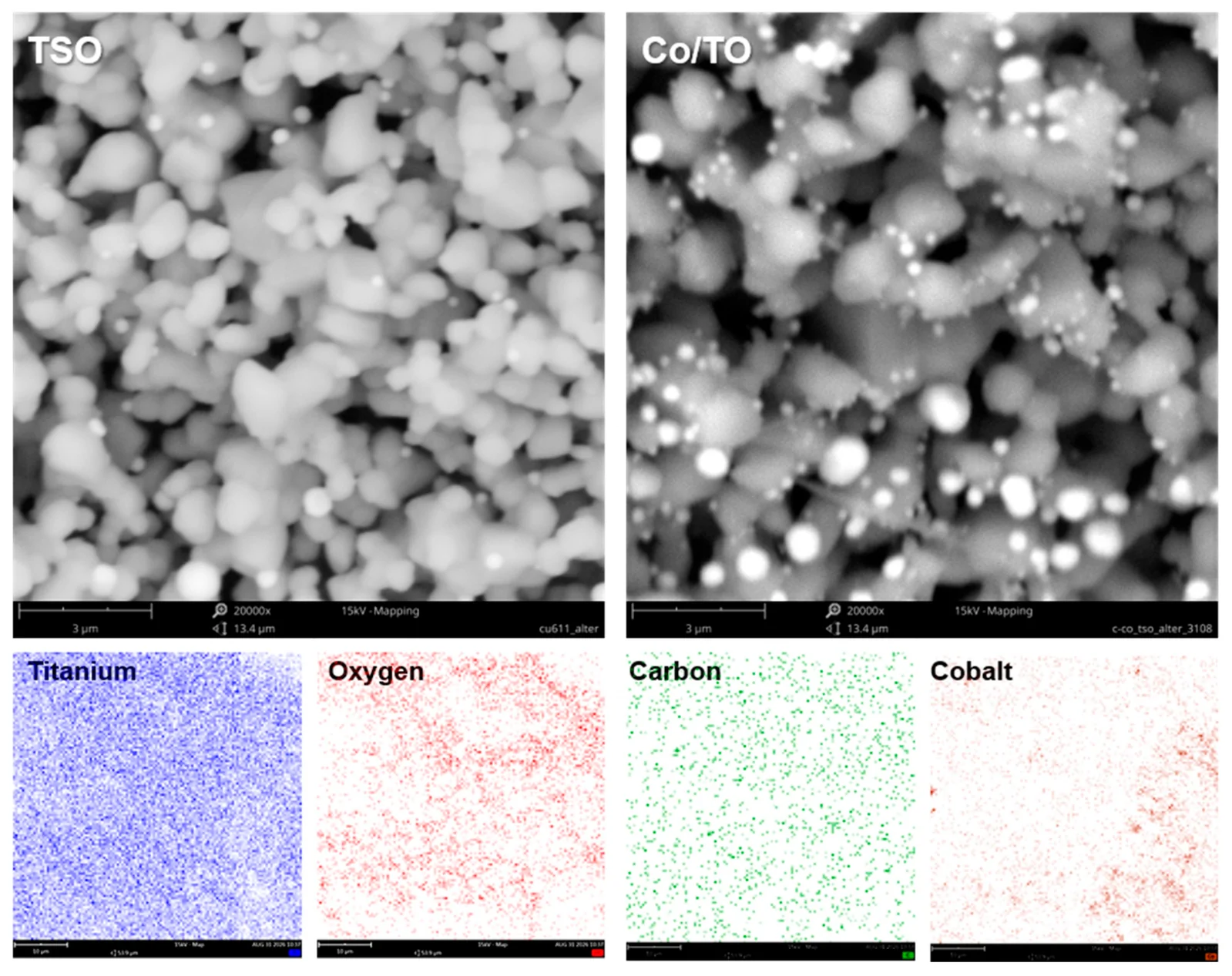
SEM images of TSO and Co/TO with elemental maps for Co/TO.

**Figure 4:**
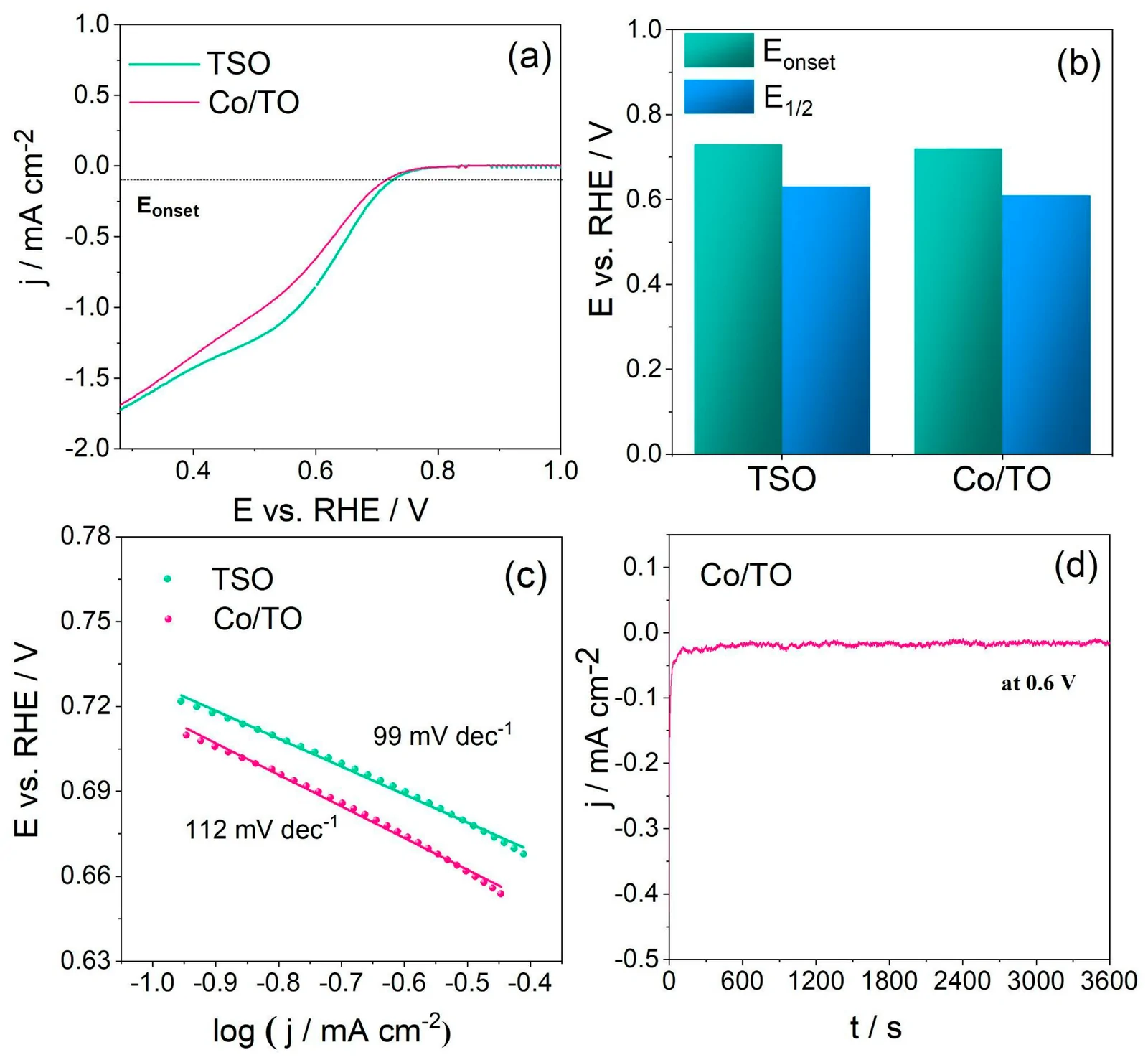
LSV RDE data at 1600 rpm and a scan rate of 20 mV s^−1^ (**a**), with E_onset_ and E_1/2_ values (**b**), and Tafel plots of TSO and Co/TO samples (**c**), and a chronoamperometric curve of Co/TO at 0.6 V (**d**).

**Figure 5:**
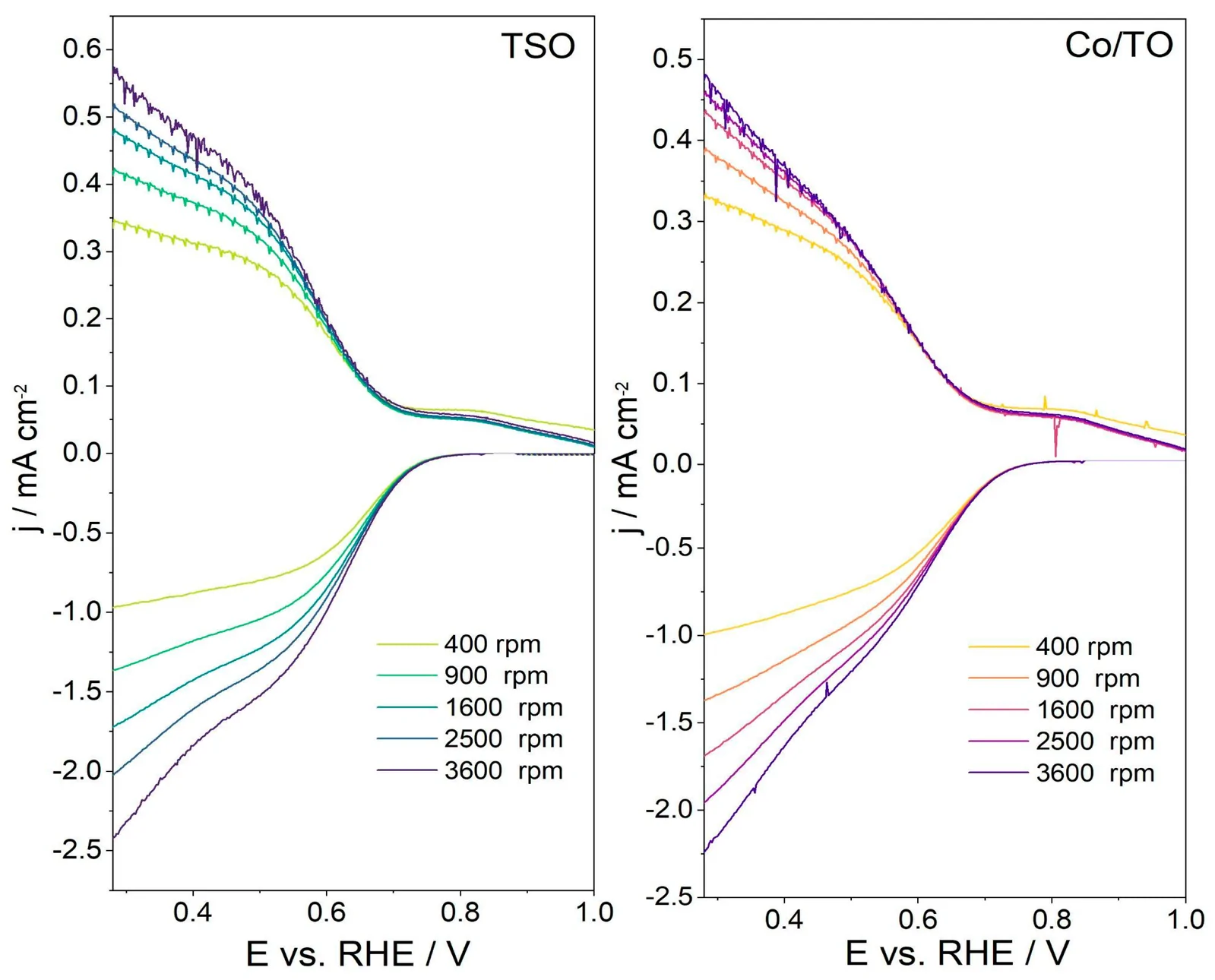
RDE investigation of oxygen reduction on the TSO and Co/TO electrode at different rotation rates: (**left**) disk current density and (**right**) ring current density associated with peroxide oxidation as functions of potential.

**Figure 6:**
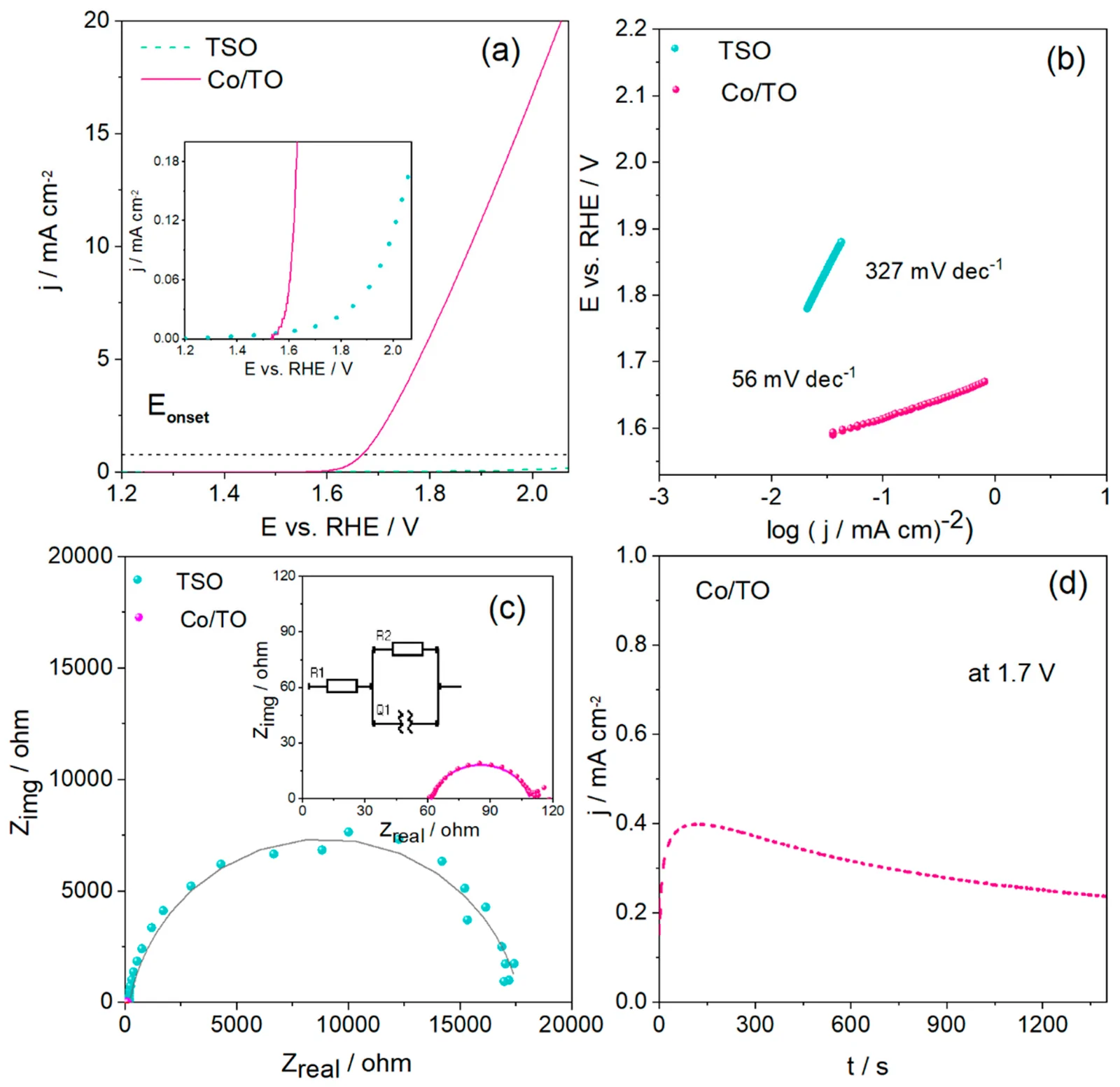
OER polarization curves at 20 mV s^−1^ and 1600 rpm (**a**), with the corresponding Tafel plots (**b**), Nyquist plots at 1.7 V of TSO and Co/TO with the corresponding equivalent circuit in the inset (**c**), and chronoamperometric curve of Co/TO at 1.7 V (**d**).

**Table 1:** Atomic content based on EDX of pristine TSO and Co/TO.

	TSO/at.%	Co/TO/at.%
Titanium	34.22	25.90
Oxygen	60.12	58.48
Cobalt	-	4.15
Carbon	5.51	11.47

**Table 2:** Comparison of ORR kinetic parameters of TSO and Co/TO with similar cobalt- or transition metal-based literature reports on electrocatalysts.

ORR Electrocatalysts	E_onset_/V	E_1/2_/V	b/mV dec^−1^	n_KL_	References
TSO	0.73	0.63	112	2.51–2.54	This work.
Co/TO	0.72	0.61	99	2.94–3.00	This work.
NiO/CoN PINWs	0.89	0.68	86	3.97	[59]
NiCo_2_O_4_ NWs	0.86	0.65	138	2.25	[59]
PBSCF-NF	/	0.73	98	/	[56]
PBSCF-P	/	/	102	/	[56]
FeCu/rGO	0.72	0.63	112	1.8–2.2	[57]
Co-Fe/CA-2	0.77	0.66	93	2.6–2.7	[58]
CoFe-PBA/C450	/	0.75	136.2	/	[60]

**Table 3:** Average electron-transfer numbers (*n*) for the ORR on TSO and Co/TO at different RRDE rotation rates.

Rotation Rate (rpm)	(n), TSO	H_2_O_2_ Yield, TSO (%)	(n), Co/TO	H_2_O_2_ Yield, Co/TO (%)
400	2.14	93.1	2.24	88.2
900	2.33	83.3	2.40	80.0
1600	2.35	82.6	2.42	78.8
2500	2.41	79.4	2.52	74.2
3600	2.52	73.9	2.60	69.9

## Data Availability

The original data presented in the study are openly available in Zenodo repository at https://doi.org/10.5281/zenodo.22303559.

## Supplementary Materials

The following supporting information can be downloaded at https://www.mdpi.com/article/10.3390/ma19194099/s1, Figure S1: CVs of TSO and Co/TO and the corresponding double-layer capacitance plots in an N_2_-saturated 1 M KOH solution; Figure S2. CVs of TSO (a) and Co/TO (b) in N_2_- and O_2_-saturated solutions.

## Abbreviations

The following abbreviations are used in this manuscript:

MP	Magnéli phase
ORR	Oxygen reduction reaction
OER	Oxygen evolution reaction
TSO	Titanium suboxides
