# Supplementary material for: Cobalt-Induced Magnéli Phase Transformation Towards Oxygen Evolution and Oxygen Reduction Reactions
Source: Materials (Basel). 2026 Sep 25;19(19):4099. doi: 10.3390/ma19194099 (PMC13665825; doi:10.3390/ma19194099)
Supplement: Supplementary file 1 [file materials-19-04099-s001.zip › materials-4576645-supplementary.pdf]

Supporting information

Cobalt-induced Magnéli phase modification towards oxygen evolution and oxygen reduction reactions

Tijana Milivojević[1], Maja Ranković[2], Jadranka Milikić[2], Stevan Stojadinović[3], Nemanja Gavrilov[2], Bojana Nedić Vasiljević[2], Danica Bajuk-Bogdanović[2], Maja Milojević-Rakić[2]

[1] Centre for Ecotoxicological Research, Bulevar Šarla de Gola 2, 81000 Podgorica, Montenegro
[2] University of Belgrade-Faculty of Physical Chemistry, Studentski Trg 12-16, 11158 Belgrade, Serbia
[3] University of Belgrade-Faculty of Physics, Studentski trg 12-16, 11158 Belgrade, Serbia

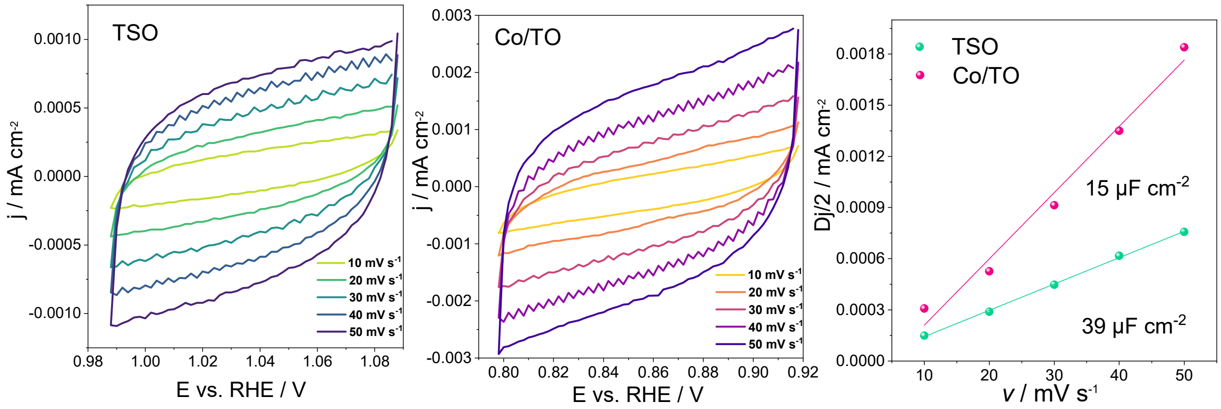

Figure S1. CVs of TSO and Co/TO and the corresponding double-layer capacitance plots in an N_2-saturated 1 M KOH solution.

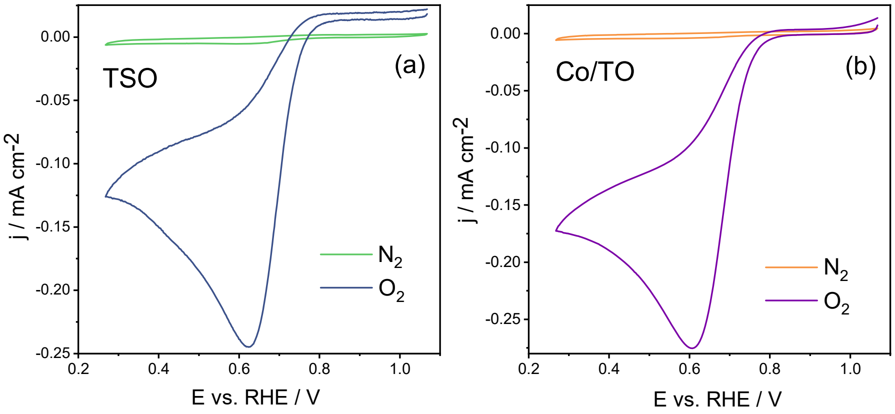

Figure S2. CVs of TSO (a) and Co/TO (b) in N_2- and O_2-saturated solutions.
